# Some *In Vitro/In Vivo* Chemically-Induced Experimental Models of Liver Oxidative Stress in Rats

**DOI:** 10.1155/2014/706302

**Published:** 2014-01-16

**Authors:** Rumyana Simeonova, Magdalena Kondeva-Burdina, Vessela Vitcheva, Mitka Mitcheva

**Affiliations:** Laboratory of Drug Metabolism and Drug Toxicity, Department of Pharmacology, Pharmacotherapy and Toxicology, Faculty of Pharmacy, Medical University 2 Dunav Street, 1000 Sofia, Bulgaria

## Abstract

Oxidative stress is critically involved in a variety of diseases. Reactive oxygen species (ROS) are highly toxic molecules that are generated during the body's metabolic reactions and can react with and damage some cellular molecules such as lipids, proteins, or DNA. Liver is an important target of the oxidative stress because of its exposure to various prooxidant toxic compounds as well as of its metabolic function and ability to transform some xenobiotics to reactive toxic metabolites (as ROS). To investigate the processes of liver injuries and especially liver oxidative damages there are many experimental models, some of which we discuss further.

## 1. Introduction

Oxidative stress is an imbalance between the production and scavenging of reactive oxygen and nitrogen species (ROS and RNS) and free radicals that can induce lipid peroxidation, DNA fragmentation, and protein oxidation [1]. These damages result in the loss of membrane integrity, structural and functional changes in proteins, and gene mutations [2]. Normally, the affected cells are trying to neutralise reactive molecules by deploying their antioxidative defense that include reduced glutathione (GSH), alpha-tocopherol, ascorbic acid, antioxidant enzymes catalase (CAT), superoxide dismutase (SOD), glutathione peroxidase (GPx), glutathione reductase (GR), and glutathione-S-transferase (GST).

Oxidative stress is critically involved in a variety of diseases. ROS are highly dangerous molecules that are generated during the body's metabolic reactions and can react with and damage some cellular molecules such as lipids, proteins, or DNA.

Liver plays a pivotal role in the regulation of various physiological processes in the body such as carbohydrate metabolism and storage, fat metabolism, bile acid synthesis, and so forth besides being the most important organ involved in the detoxification of various drugs as well as xenobiotics in our body [3].

It is highly susceptible to damage by xenobiotics owing to its continuous exposure to these toxicants via the portal blood circulation [4]. Various chemicals, like carbon tetrachloride (CCl_4_), tert-butyl hydroperoxide (t-BHP), alcohol, paracetamol, galactosamine (GalN), and others, can cause potential damage to the liver cells leading to progressive dysfunction. Most of the hepatotoxic chemicals cause damage to the hepatocytes by inducing lipid peroxidation [5, 6]. Thus, the disorders associated with liver are numerous and varied.

One of the most important liver toxicity mechanisms might be a consequence of cell damage by ROS and RNS. Kupffer cells release reactive oxygen species (ROS), cytokines, and chemokines, which induce neutrophil extravasation and activation. Also the liver expresses many cytochrome P450 isoforms, including ethanol-induced CYP2E1. CYP2E1 generates ROS, activates many toxicologically important substrates, and may be the central pathway by which some substances cause oxidative stress (ethanol, carbon tetrachloride, etc.) [7].

In this review we summarize some commonly used toxic models employed in the study of hepatotoxicity and hepatoprotection. A number of models of hepatic disorders support the notion that ROS have a causal role in liver injuries. Experimental liver injuries are induced by specific toxic compounds, because the formation of ROS is stimulated by a number of xenobiotics.

## 2. Carbon Tetrachloride (CCl_4_)

Carbon tetrachloride (CCl_4_) is the most widely used model to develop oxidative stress and liver toxicity in rats. Hepatic injury through carbon tetrachloride induced lipid peroxidation is well known and has been extensively used in the experimental models to understand the cellular mechanisms behind oxidative damage and further to evaluate the therapeutic potential of drugs and dietary antioxidants [8].

CCl_4_ is activated by cytochrome CYP2E1, CYP2B1, or CYP2B2, and possibly CYP3A, to form the trichloromethyl radical, CCl3* [9]. This radical can bind to cellular molecules (nucleic acid, protein, lipid), impairing crucial cellular processes such as lipid metabolism, with the potential outcome of fatty degeneration (steatosis) [10]. This radical can also react with oxygen to form the trichloromethylperoxy radical CCl_3_OO*, a highly reactive species. CCl_3_OO* initiates the chain reaction of lipid peroxidation, which attacks and destroys polyunsaturated fatty acids [9]. Among the degradation products of fatty acids are reactive aldehydes, malondialdehyde (MDA), and 4-hydroxynonenal, which bind easily to functional groups of proteins and inhibit important enzyme activities. Disturbed cellular processes are most likely due to increased levels of these thiobarbituric acid reactive species (TBARS) [11], lactate dehydrogenase (LDH) leakage as a result of membrane breakdown and concomitant increase in membrane permeability [12], loss of cell protection, witnessed by GSH depletion and as a result of all these changes—cell death.

In our laboratory we use some *in vitro *and* in vivo* hepatotoxicity models based on CCl_4_-induced liver damage in Wistar rats and in spontaneously hypertensive rats (SHR). *In vitro* experiments are carried out in primary isolated rat hepatocytes [13] or liver microsomes [14]. Cell incubation with CCl_4_ (86 *μ*mol L^−1^) leads to a significant decrease in cell viability, increased LDH leakage, decreased levels of cellular GSH, and elevation in MDA quantity. Enzyme-induced LPO is started with 20 mM CCl_4_ in the presence of 1 mM NADPH [14]. For *in vivo* experiments Wistar rats are challenged with a single dose (2 mL/kg) of 20% of CCl_4_ in olive oil [15]. These *in vitro/in vivo* CCl_4_-induced liver injury models are useful for investigations on hepatoprotective and antioxidant properties of some plant-derived biologically active compounds [13–17].

We found that ROS, produced by CCl_4_, decrease the activities not only of antioxidant enzymes such as catalase (CAT), superoxide dismutase (SOD), glutathione peroxidase (GPx), glutathione reductase (GR), and glutathione-S-transferase (GST) [18], but also the activities of some drug metabolizing enzymes such as CYP2E1 and CYP3A, involved in their production [15].

## 3. Tert-Butyl Hydroperoxide (t-BHP)

The cellular system of energy supply localized in mitochondria is another target of many hepatotoxic substances causing oxidative stress and is one of the most important mechanisms through which hepatotoxic factors induced apoptotic and necrotic processes [19].

Tert-butyl hydroperoxide caused necrosis through inducing mitochondrial reactive oxygen formation [20]. As a prooxidant, t-BHP was widely used and many effects on cell metabolism have been described, for example, changes in calcium homeostasis [21], increase of lipid peroxidation, or decrease of mitochondrial membrane potential [22, 23]. Two mechanisms for t-BHP action were proposed: depletion of cellular stores of GSH and oxidation of functionally important SH groups on mitochondrial enzymes [24] and/or changes of mitochondrial membrane integrity induced by peroxidation of membrane lipids [22, 23]. The metabolism of t-BHP to free radicals undergoes through several steps. In microsomal suspension, in the absence of NADPH, it has been shown to undergo one-electron oxidation to a peroxyl radical (1), whereas in the presence of NADPH it has been shown to undergo one-electron reduction to an alkoxyl radical (2). In isolated mitochondria and intact cells, the t-BHP has been shown to undergo *β*-scission to the methyl radical (3). All these radicals cause lipid peroxidation process [25, 26]:

(1) $$\begin{array}{c} {\left(\text{C}{\text{H}}_{3}\right)}_{3}\text{COOH}⟶{\left(\text{C}{\text{H}}_{3}\right)}_{3}\text{CO}{\text{O}}^{•}+{\text{e}}^{-}+{\text{H}}^{+} \end{array}$$

(2) $$\begin{array}{c} {\left(\text{C}{\text{H}}_{3}\right)}_{3}\text{COOH}+{\text{e}}^{-}27\text{F}6{\left(\text{C}{\text{H}}_{3}\right)}_{3}\text{C}{\text{O}}^{•}+\text{O}{\text{H}}^{-} \end{array}$$

(3) (CH3)3CO•⟶(CH3)2CO+CH3•

Experiments on isolated hepatocytes are thus a useful model system for evaluation of the toxic effect of various prooxidants which act directly on mitochondrial enzymes. In our experiments using freshly isolated rat hepatocytes we found that t-BHP (75 *μ*mol L^−1^) decreases cell viability [27, 28]. It causes leakage of lactate dehydrogenase (LDH) and formation of malondialdehyde in hepatocytes. Furthermore, t-BHP causes the depletion of cellular GSH levels. These data correlate with the results obtained by many authors [23–25].

Enhanced formation of ROS has been suggested to play a role in some liver disease processes, including alcohol-induced liver injury [29–31], paracetamol-induced liver failure [32, 33], and many others. Many other drugs as isoniazide, amiodarone, and valproic acid as well as widely used and abused substances as nicotine and cocaine damage liver cells by producing toxic ROS. Because of their widespread consumption, they are also used as experimental models of liver injuries.

## 4. Ethanol 

Acute and chronic ethanol treatments increase the production of ROS, lower cellular antioxidant levels, and enhance oxidative stress in many tissues, especially the liver. Ethanol-induced oxidative stress plays a major role in the mechanisms by which ethanol produces liver injury [34].

The liver expresses many cytochrome P450 isoforms, including ethanol-induced CYP2E1. CYP2E1 generates ROS, activates many toxicologically important substrates, and may be the central pathway by which ethanol causes oxidative stress [7].

CYP2E1 metabolizes and activates many toxicologically important substrates, including ethanol, carbon tetrachloride, acetaminophen, and N-nitrosodimethylamine, to more toxic products [35, 36]. Induction of CYP2E1 by ethanol is a central pathway by which ethanol generates oxidative stress. In our intragastric model of ethanol feeding (3 g/kg, 14 days) of spontaneously hypertensive rats (SHR) a prominent induction of CYP2E1 occurs along with significant alcohol liver injury [37]. Lipid peroxidation also occurs, and ethanol-induced liver pathology correlates with CYP2E1 levels and elevated lipid peroxidation [38]. Chronic ethanol consumption is associated with reduced liver GSH and alpha-tocopherol level and with reduced superoxide dismutase (SOD), catalase (CAT) and glutathione peroxidase (GPx) activity [39]. Our results, concerning normotensive rats (Wistar-Kyoto), are in accordance with these data, whereas alcohol intake in SHR increases significantly SOD and CAT activities by approximately 50% [37]. We suggested that the differences in antioxidant status and the effect of ethanol between the strains might be due to the oxidative stress state in the hypertensive pathological model. Additionally we found that multiple ethanol treatment resulted in less pronounced effect on the assessed parameters (MDA, GSH, nNOS) in the female SHR, compared to male SHR [38]. These results might be due to a protective effect of estrogens on the oxidative stress and to a stimulation of the antioxidant defense systems, in liver.

## 5. Paracetamol

Paracetamol (PCM) is primarily metabolized by sulfation and glucuronidation, but with an increasing dose rate; these pathways become saturated and a greater proportion of the drug is available for oxidation by the microsomal cytochrome P-450 system [40]. N-Acetyl-P-benzoquinone Imine (NAPQI) is the product of this pathway which is thought to be responsible for the subsequent hepatic damage [41]. N-acetyl-P-benzoquinone imine (NAPQI) is a highly reactive electrophile and is detoxified in liver by either reduction to the parent compound, acetaminophen, or conjugation at the metaposition with glutathione, in which both reactions consume GSH [42].

Glutathione (GSH) plays an important role in protecting cells from electrophilic compounds and free radicals such as reactive oxygen species generated during cellular metabolism. Reduced glutathione can act as a reductant, reducing hydrogen peroxide and lipid hydroperoxides directly to H_2_O, a reaction catalyzed by GSH-Px [43]. Depletion of intracellular GSH, under conditions of continuous intracellular oxidative stress, leads to oxidation and damage of lipids, proteins, and DNA by the reactive oxygen species [44, 45].

The importance of glutathione in PCM toxicity is further emphasized by the large body of evidence which indicates that interventions which increase GSH content can dramatically reduce PCM and NAPQI-induced hepatic injury [46, 47]. Our previous studies have shown that GSH content in animal livers decreases after PCM overdose (2 g/kg, i.p. single dose) [15] and have proved that biologically active compounds derived from plants are useful for treatment of PCM-induced liver disorders, because of a stimulation of GSH synthesis.

Oxidative stress is also considered to be involved in the induction of hepatotoxicity by PCM. Oxidation of PCM by CYPs may generate ROS. Hydrogen peroxide and superoxide are produced during metabolic activation of PCM in the mixed function oxidase system [47].

## 6. Nicotine

During smoking, nicotine is rapidly absorbed into the circulatory system where more than 80% is metabolized in the liver [48]. Liver is an important organ and is responsible for biotransformation of drugs and other toxins to remove them from the body. Nicotine from heavy smoking increases the risk of developing some dangerous liver disorders by one of the main mechanisms being the oxidative stress. Increased production of free radicals or decreased function of the defense system play an important role in nicotine toxicity [49]. Also maternal nicotine exposure induces oxidative stress and causes histopathological changes in the lung and liver of lactating offspring [50]. Nicotine induces oxidative stress both *in vivo* and *in vitro* that causes a peroxidant/antioxidant imbalance in blood cells, blood plasma, and other tissues [51]. Some authors [50, 52] reported that nicotine induces oxidative stress and depleted antioxidant defense mechanisms through reduction of glutathione peroxidase in liver, lung, and kidney. Oxidative stress generates free radicals that attack the membrane lipids resulting in the formation of malondialdehyde (MDA), which causes peroxidative tissue damage [53]. Animal's studies have shown significantly higher liver and serum levels of MDA, conjugated dienes, hydroperoxides, and free fatty acids in rats intoxicated by nicotine [54, 55].

Nicotine is not recognized as a common experimental model for liver injuries, but because of its well-established prooxidant mechanisms of hepatotoxicity, and widespread consumption is used from many authors [51, 53, 55, 56] for investigations of antioxidant and protective properties of natural compounds.

In our previous experiments [57, 58] enhanced level of tissue lipid peroxides in nicotine treated rats (1 mg/kg i.p; 6,5 mg/kg p.o.) has been shown to be accompanied by a significant decrease in the levels of GSH, glutathione peroxidase (GPx), superoxide dismutase (SOD), and catalase (CAT) and increased glutathione reductase (GR) activity in Wistar rat liver.

## 7. D-Galactosamine (GAL)

D-Galactosamine is a well-known experimental hepatotoxin usually used to produce acute toxicity in rat liver. Galactosamine metabolism depletes the uridine pool of hepatocytes, thus inducing transcriptional arrest and causing an increase in sensitization to cytokines such as TNF-*α* and an increase in oxidative stress and GSH depletion, which lead to mitochondrial dysfunction and cell death [59]. Both oxidative and nitrosative stress play a key role in the pathogenesis of GAL-induced hepatic injury [60].

Usually rats are injected (i.p.) with GAL (400 mg/kg b.w.) as a single dose [61].

## 8. Cocaine 

Cocaine is a psychoactive drug that has been recognized as one of the most significant examples of drug abuse. Its misuse can induce severe toxic effects, including neurotoxicity, cardiotoxicity, and hepatotoxicity. One of the main mechanisms discussed for cocaine-induced liver injury is promotion of lipid peroxidation by ROS which are produced during cocaine bioactivation to norcocaine through N-demethylation by cytochrome P 450 and flavin adenine dinucleotide containing monooxygenases [62].

A large body of evidence in both human and experimental models suggests that impairment of the antioxidant defense system by cocaine and its metabolites plays a role in the pathogenesis of cocaine hepatotoxicity [62–64]. In particular, glutathione seems to play an important protective role against cocaine-induced hepatic injury. For example, the acute administration of cocaine to rats [65] and multiple treatments of mice [63] have been shown to deplete the cellular reduced glutathione, to decrease the activity of superoxide dismutase (SOD), catalase (CAT), and glutathione peroxidase (GPx) and to increase glutathione reductase (GR) activity. The GSH depletion, induced by cocaine administration, observed in these and other studies [62, 66] might be explained by increased utilization of GSH for detoxification of ROS and lipid peroxidation products. The critical role of ROS and oxidative stress in the pathogenesis of cocaine-induced liver damage was demonstrated by the observed ameliorating effects of bioactive compounds with an antioxidant activity, administered several days before cocaine treatment [65, 67]. The bioactive compounds were found to decrease cocaine toxicity both by increasing GSH levels and antioxidant enzyme activities.
